# Unexpected resolution of chronic gastroesophageal reflux disease with testosterone replacement therapy

**DOI:** 10.1210/jcemcr/luag082

**Published:** 2026-05-05

**Authors:** Rupen Tamang, Benjamin Disney, Jeff Foster

**Affiliations:** Voy, Menwell Ltd, UK; University Hospitals Coventry and Warwickshire NHS Trust, UK; Croft Medical Centre NHS GP, UK; British Society of Sexual Medicine, UK

**Keywords:** gastroesophageal reflux disease (GERD), testosterone replacement therapy (TRT), hypogonadism, proton pump inhibitor (PPI)

## Abstract

A 63-year-old man with a chronic, 30-year, history of gastroesophageal reflux disease (GERD) that required long-term proton pump inhibitor (PPI) use experienced complete symptom resolution after initiation of testosterone replacement therapy (TRT) for symptomatic hypogonadism. After 11 months of TRT, the patient inadvertently discontinued PPI therapy (esomeprazole 20 mg once daily) while away on vacation without any of the expected rebound acid hypersecretion he had previously experienced in the past when trying to stop. Three months post-PPI cessation, he remained asymptomatic with no requirement for antireflux medication. This case presents a novel and unexpected beneficial effect of TRT on the symptoms of GERD and contrasts with current literature that suggest hormone replacement therapy, with estrogen and progesterone, typically exacerbates reflux disease. Mechanisms behind this improvement may involve anti-inflammatory properties of testosterone, a direct effect on lower esophageal sphincter tone and improved gastric motility, or indirect benefits through weight loss and increased physical activity. Or, as is most likely, a combination of several factors. This paper highlights the need for further investigation into the relationship between testosterone and gastrointestinal physiology in men with hypogonadism.

## Introduction

Gastroesophageal reflux disease (GERD) affects approximately 14% of the world's population and represents a substantial burden on health-care systems and patients’ quality of life [1]. Long-term management of this condition typically involves a proton pump inhibitor (PPI), though their prolonged use is associated with potential adverse health risks that include bone fractures, micronutrient deficiencies, and increased risk of enteric infections such as *Clostridioides difficile* [2].

The British Society of Sexual Medicine (BSSM) defines male hypogonadism as a clinical and biochemical syndrome caused by a dysfunction of the hypothalamic-pituitary-gonadal axis, that combines specific symptoms in the presence of low serum testosterone levels, confirmed by at least 2 morning samples [3]. With a prevalence of 38.7% in men aged 45 and older, it is estimated that male hypogonadism affects 2.4 million men between ages 40 and 69 years in the United States [4, 5]. Furthermore, testosterone replacement therapy (TRT) has been demonstrated to have a range of benefits in men with testosterone deficiency that include improved sexual function, increased muscle mass, reduced risk of osteoporosis, enhanced mood, and increased energy levels [6]. The relationship between TRT and gastrointestinal conditions, specifically GERD, however, has not been extensively studied.

The relationship between sex-steroid hormones and GERD presents an intriguing paradox. Current literature predominantly demonstrates that exogenous hormone replacement therapy—particularly estrogen and progesterone—increases GERD risk in women [7, 8]. Epidemiological studies in men have shown a positive relationship between endogenous testosterone and Barrett esophagus with a suggestion that androgens may promote esophageal pathology [9]. However, a retrospective study of Japanese men indicated a negative correlation between testosterone levels and severity of GERD symptoms [10]. Data on the effects of therapeutic testosterone supplementation on the symptoms of GERD in hypogonadal men remain scarce.

## Case presentation

A 63-year-old, semi-retired, White British man presented with symptoms of testosterone deficiency in September 2024. His main symptoms included profound fatigue, sexual dysfunction (which included a complete loss of libido and absence of morning erections), persistent muscle aches, and a reduction in his motivation along with enthusiasm for life. This consequently led to low mood and weight gain of 7 kg, which increased his baseline body mass index (BMI) from 23.9 (82 kg) to 26.0 (89 kg).

Past medical history included persistent GERD and well-controlled hypertension. Initially diagnosed with a *Helicobacter pylori* infection that was treated successfully with eradication therapy, the patient's reflux symptoms developed post eradication and were controlled only by consistent, long-term PPI use, of which he had been on esomeprazole 20 mg daily for the past 8 years. If the patient stopped this treatment for a few days, reflux symptoms would return, which was described as a severe, daily, substernal burning pain that radiated to the oropharynx. Nocturnal reflux symptoms disrupted sleep up to 3 times a week and were alleviated by Gaviscon (Reckitt Benckiser Healthcare (UK) Limited). Gastroscopy showed no evidence of Barrett esophagus, erosive esophagitis, or structural abnormality. In terms of lifestyle factors, the patient was a nonsmoker and consumed 3 units of alcohol per week. This, in addition to a balanced diet, had a minimal effect on exacerbation of his GERD symptoms.

With his symptoms lasting for several years, the patient had initially sought evaluation through his primary care physician in 2022. At that time, his morning total testosterone level was 317 ng/dL (SI: 11 nmol/L). The BSSM advises that men with serum levels of total testosterone less than 230 ng/dL (SI: 8 nmol/L) and symptoms consistent with testosterone deficiency have a strong indication for TRT, while men with a total testosterone of up to 346 ng/dL (SI: 12 nmol/L) should be considered for a trial of TRT if symptomatic [11, 12]. In addition, following publication of the testosterone treatment to prevent or revert type 2 diabetes in men enrolled in a lifestyle program (T4DM) study in 2021, patients could also be considered for a trial of TRT if their total testosterone is less than 433 ng/dL (SI: 15 nmol/L), they are symptomatic, and have a metabolic syndrome such as type 2 diabetes [11]. Free testosterone, a measure of bioavailable testosterone, and a useful additional indicator in the diagnosis of male hypogonadism, was not tested for at the time of the patient's first evaluation in primary care. Due to restrictions in local prescribing guidance, and limitations of regional reference ranges, the patient was not offered treatment, and reassured that his blood results were normal.

## Diagnostic assessment

Two and half years on from his initial assessment, the patient repeated his hormone profile blood tests, which revealed a repeat total testosterone on 2 occasions of 377 ng/dL (SI: 13 nmol/L), a luteinizing hormone of 13.8 mIU/mL (SI: 13.8 IU/L) (reference range, 1.6-8.0 mIU/mL), follicle-stimulating hormone of 64.7 mIU/mL (SI: 64.7 IU/L) (reference range, 1.3-8.4 mIU/mL), and sex hormone–binding globulin of 1140 ng/dL (SI: 39.6 nmol/L) (reference range, 288-2019ng/dL; 10-70 nmol/L). In the absence of metabolic syndrome or type 2 diabetes, this would have placed the patient's results within the normal range, but with an albumin level of 50 g/L (reference range, 35-50 g/L), subsequent calculation of his free testosterone levels resulted in a level of 6.32 ng/dL (SI: 0.219 nmol/L). The BSSM advises that patients with symptoms and a free testosterone of less than 6.49 ng/dL (SI: 0.225 nmol/L) may also be a potential candidate for a trial of TRT. Therefore, due to the chronicity of his symptoms, combined with his low free testosterone result, the patient agreed to a 6-month trial of TRT.

## Treatment

TRT was initiated in December 2024 with weekly injections of testosterone cypionate 200 mg/mL, titrated to a weekly maintenance dose of 40 mg (0.2 mL). Blood tests, performed as per the BSSM guidance at 3 and 6 months, showed a rise in testosterone levels back to normal levels with a mean average result of 690 ng/dL (SI: 23.9 nmol/L), the optimal range being between 433 and 865 ng/dL (SI: 15-30 nmol/L) [3].

## Outcome and follow-up

The patient continued with doxazosin 4 mg daily for hypertension. Inadvertent cessation of his PPI occurred in October 2025, while on vacation. With no symptom recurrence, this led to discontinuation of esomeprazole and Gaviscon.

## Discussion

This case presents the unexpected, but clinically significant, resolution of severe persistent GERD after initiation of TRT in a 63-year-old man with hypogonadism. Complete symptom resolution allowed for safe cessation of PPI therapy after 8 years, with no rebound acid hypersecretion, and eliminated the well-documented risks of long-term PPI use.

TRT may have a beneficial gastrointestinal effect in this population, potentially through mechanisms distinct from those that operate in eugonadal individuals or female hormone replacement therapy. The potential clinical effect is substantial. Thirty years of GERD had significantly impaired this patient's quality of life, which had now been ameliorated.

Androgens may modulate lower esophageal physiology through several pathways. Testosterone could increase the resting tone, rescue the frequency of transient relaxations, or improve contractility of the esophagus.

Animal studies have demonstrated sex-steroid hormone receptors are present in esophageal tissue, which indicates that a direct hormonal modulation is biologically plausible [13]. While some literature suggests testosterone may increase reflux propensity by affecting lower esophageal tone, the net effect in hypogonadal men being optimized to physiological levels may differ from pharmacological androgen exposure in eugonadal individuals [9].

Testosterone exhibits anti-inflammatory properties, through a reduction in proinflammatory cytokines (interleukin [IL]-1β, IL-6, and tumor necrosis factor-α) [14, 15]. GERD pathophysiology involves inflammation due to lower esophageal sphincter dysfunction, which leads to an impaired esophageal clearance, delayed gastric emptying, and increased intra-abdominal pressure [16]. Testosterone-mediated reduction in systemic and local esophageal inflammation could decrease symptom severity, improve mucosal integrity, and raise the threshold for symptomatic reflux.

TRT assisted with weight loss, and resulted in a 7-kg reduction in body weight, which decreased the patient’s BMI from 26.0 (89 kg) to 23.9 (82 kg). Weight loss, an established intervention for GERD, leads to lower intra-abdominal pressure, decreased gastroesophageal pressure gradient, and reduction in transient lower esophageal sphincter relaxation [17]. However, this patient had previously maintained a baseline weight of 82 kg while still symptomatic of severe GERD, suggesting weight loss alone is insufficient as an explanation.

More important, testosterone selectively reduces visceral adipose tissue with preservation or increase in lean muscle mass [18]. Visceral adiposity contributes more significantly to intra-abdominal pressure and GERD risk than total body weight [19]. Testosterone-induced visceral fat reduction may provide more benefits beyond simple weight loss as explained by the dramatic symptom improvement despite modest weight loss.

Resolution of reflux symptoms that occurred 11 months after TRT initiation demonstrates a clear temporal sequence. However, the long latency period raises questions about direct causality vs delayed indirect effects, such as cumulative weight loss and body composition changes.

Although asking about reflux symptoms is not standard practice for a TRT follow-up consultation, in this case, the patient voluntarily discussed the improvement in his symptoms. A clear dose-response relationship was not evident as symptom resolution persisted despite titration down to 40 mg weekly with testosterone levels sustained within the therapeutic range. This may indicate that improvement in symptoms of GERD is related to the establishment of physiological testosterone levels. The improvement appeared specific to GERD, and this specificity suggests a targeted effect on esophagogastric physiology rather than nonspecific enhancement of general well-being.

Reversibility could not be assessed as TRT was not discontinued. While recurrence of GERD with treatment cessation would provide stronger causal evidence, such an intervention would not be appropriate given the patient's clinical improvement.

Alternative explanations for symptom resolution were considered unlikely. Neither the patient's weight loss nor increased physical activity targeted GERD management as he remained symptomatic at similar past weights. A placebo effect and spontaneous remission were also improbable given the unexpected, sustained improvement after PPI cessation and the condition's severe chronicity.

This case highlights a potential link between the treatment of testosterone deficiency in men, and subsequent improvements in GERD. A causal relationship between TRT and GERD resolution is plausible, but not yet definitively established. Possible mechanisms of action include the direct effects of testosterone on esophagogastric physiology, visceral fat reduction, increased physical activity, and improved overall physiological function. However, weight loss alone does not appear sufficient to explain the magnitude of response in this case, given the patient's past medical history. Overall, this report highlights how much is still unknown about how testosterone affects our physiology, and may have an effect on various comorbidities. Further studies are needed to establish a definitive link between the two conditions.

## Learning points

- Beneficial effects of TRT may extend beyond classic hypogonadal symptoms.
- Complete resolution of severe, long-standing GERD occurred in a patient on TRT.
- Multiple mechanisms may contribute to improvement of GERD symptoms with TRT.

## Contributors

All authors made individual contributions to authorship. J.F. was involved in the initial diagnosis and management of the patient. R.T. was involved in data collection through an interview with the patient to establish details of his journey. B.D. contributed to the clinical interpretation of the gastroenterological aspects of the case and to the development of the background and discussion sections. J.F., R.T., and B.D. all reviewed and approved the final draft.

## Data Availability

Some or all datasets generated during and/or analyzed during the current study are not publicly available but are available from the corresponding author on reasonable request.

## Abbreviations

BMI: body mass index
BSSM: British Society of Sexual Medicine
GERD: gastroesophageal reflux disease
PPI: proton pump inhibitor
TRT: testosterone replacement therapy

## References

[1] Nirwan JS, Hasan SS, Babar ZUD, Conway BR, Ghori MU. Global prevalence and risk factors of Gastro-oesophageal Reflux Disease (GORD): systematic review with meta-analysis. Sci Rep. 2020;10(1):5814. 32242117 10.1038/s41598-020-62795-1 PMC7118109

[2] Freedberg DE, Kim LS, Yang YX. The risks and benefits of long-term use of proton pump inhibitors: expert review and best practice advice from the American gastroenterological association. Gastroenterology. 2017;152(4):706‐715. 28257716 10.1053/j.gastro.2017.01.031

[3] Hackett G, Kirby M, Rees R, et al The British society for sexual medicine guidelines on male adult testosterone deficiency, with statements for practice. World J Mens Health. 2023;41(3):508‐537. 36876744 10.5534/wjmh.221027 PMC10307648

[4] Mulligan T, Frick MF, Zuraw QC, Stemhagen A, Mcwhirter C. Prevalence of hypogonadism in males aged at least 45 years: the HIM study. Int J Clin Pract. 2006;60(7):762‐769. 16846397 10.1111/j.1742-1241.2006.00992.x PMC1569444

[5] Araujo AB, O’Donnell AB, Brambilla DJ, et al Prevalence and incidence of androgen deficiency in middle-aged and older men: estimates from the Massachusetts male aging study. J Clin Endocrinol Metab. 2004;89(12):5920‐5926. 15579737 10.1210/jc.2003-031719

[6] Corona G, Goulis DG, Huhtaniemi I, et al European Academy of Andrology (EAA) guidelines on investigation, treatment and monitoring of functional hypogonadism in males. Andrology. 2020;8(5):970‐987. 32026626 10.1111/andr.12770

[7] Jacobson BC, Moy B, Colditz GA, Fuchs CS. Postmenopausal hormone use and symptoms of Gastroesophageal reflux. Arch Intern Med. 2008;168(16):1798. 18779468 10.1001/archinte.168.16.1798 PMC2761884

[8] Close H, Mason JM, Wilson D, Hungin APS. Hormone replacement therapy is associated with gastro-oesophageal reflux disease: a retrospective cohort study. BMC Gastroenterol. 2012;12(1):56. 22642788 10.1186/1471-230X-12-56 PMC3411455

[9] Cook MB, Wood SN, Cash BD, et al Association between circulating levels of sex steroid hormones and Barrett's Esophagus in men: a case–control analysis. Clin Gastroenterol Hepatol. 2015;13(4):673‐682. 25158929 10.1016/j.cgh.2014.08.027 PMC4339666

[10] Harada K, Hanayama Y, Yasuda M, et al Clinical relevance of low androgen to gastroesophageal reflux symptoms. Endocr J. 2018;65(10):1039‐1047. 30068893 10.1507/endocrj.EJ18-0187

[11] Wittert G, Bracken K, Robledo KP, et al Testosterone treatment to prevent or revert type 2 diabetes in men enrolled in a lifestyle programme (T4DM): a randomised, double-blind, placebo-controlled, 2-year, phase 3b trial. Lancet Diabetes Endocrinol. 2021;9(1):32‐45. 33338415 10.1016/S2213-8587(20)30367-3

[12] Bhasin S, Brito JP, Cunningham GR, et al Testosterone therapy in men with Hypogonadism: an endocrine society* clinical practice guideline. J Clin Endocrinol Metab. 2018;103(5):1715‐1744. 29562364 10.1210/jc.2018-00229

[13] Chen TS, Doong ML, Chang FY, Lee SD, Wang PS. Effects of sex steroid hormones on gastric emptying and gastrointestinal transit in rats. Am J Physiol Gastrointest Liver Physiol. 1995;268(1):G171‐G176. 10.1152/ajpgi.1995.268.1.G171 7840200

[14] Mohamad NV, Wong SK, Wan Hasan WN, et al The relationship between circulating testosterone and inflammatory cytokines in men. Aging Male. 2018;22(2):129‐140. 29925283 10.1080/13685538.2018.1482487

[15] Bianchi VE . The anti-inflammatory effects of testosterone. J Endocr Soc. 2018;3(1):91‐107. 30582096 10.1210/js.2018-00186 PMC6299269

[16] Tack J, Pandolfino JE. Pathophysiology of gastroesophageal reflux disease. Gastroenterology. 2018;154(2):277‐288. 29037470 10.1053/j.gastro.2017.09.047

[17] El-Serag H . Role of obesity in GORD-related disorders. Gut. 2008;57(3):281‐284. 18268049 10.1136/gut.2007.127878

[18] Saad F, Aversa A, Isidori AM, Gooren LJ. Testosterone as potential effective therapy in treatment of obesity in men with testosterone deficiency: a review. Curr Diabetes Rev. 2012;8(2):131‐143. 22268394 10.2174/157339912799424573 PMC3296126

[19] Kelly DM, Jones TH. Testosterone and obesity. Obes Rev. 2015;16(7):581‐606. 25982085 10.1111/obr.12282

